# Antagonistic pleiotropy and the evolution of extraordinary lifespans in eusocial organisms

**DOI:** 10.1002/evl3.230

**Published:** 2021-05-17

**Authors:** Jan J. Kreider, Ido Pen, Boris H. Kramer

**Affiliations:** ^1^ Theoretical Research in Evolutionary Life Sciences, Groningen Institute for Evolutionary Life Sciences University of Groningen Nijenborgh 7 Groningen 9747 AG The Netherlands

**Keywords:** ageing, antagonistic pleiotropy, caste antagonism, eusociality, indirect genetic effects, individual‐based simulations, intralocus conflict, life history evolution, senescence, social insects

## Abstract

Queens of eusocial species live extraordinarily long compared to their workers. So far, it has been argued that these lifespan divergences are readily explained by the classical evolutionary theory of ageing. As workers predominantly perform risky tasks, such as foraging and nest defense, and queens stay in the well‐protected nests, selection against harmful genetic mutations expressed in old age should be weaker in workers than in queens due to caste differences in extrinsic mortality risk, and thus, lead to the evolution of longer queen and shorter worker lifespans. However, these arguments have not been supported by formal models. Here, we present a model for the evolution of caste‐specific ageing in social insects, based on Williams’ antagonistic pleiotropy theory of ageing. In individual‐based simulations, we assume that mutations with antagonistic fitness effects can act within castes, that is, mutations in early life are accompanied by an antagonistic effect acting in later life, or between castes, where antagonistic effects emerge due to caste antagonism or indirect genetic effects between castes. In monogynous social insect species with sterile workers, large lifespan divergences between castes evolved under all different scenarios of antagonistic effects, but regardless of the degree of caste‐specific extrinsic mortality. Mutations with antagonistic fitness effects within castes reduced lifespans of both castes, while mutations with between‐caste antagonistic effects decreased worker lifespans more than queen lifespans, and consequently increased lifespan divergences. Our results challenge the central explanatory role of extrinsic mortality for caste‐specific ageing in eusocial organisms and suggest that antagonistic pleiotropy affects castes differently due to reproductive monopolization by queens, hence, reproductive division of labor. Finally, these findings provide new insights into the evolution of tissue‐specific ageing in multicellular organisms in general.

**Impact Summary**

The basic principles of why organisms age are well explained by the classical evolutionary theory of ageing. However, no attempts have been made to quantitatively predict intraspecific variation in senescence because most species exhibit only little variation in lifespan for which the models are not subtle enough. As lifespans of castes in eusocial species can vary by several orders of magnitude, eusocial organisms—such as termites, ants, and some bees and wasps—are excellent model organisms to develop more quantitative theory for the evolution of ageing. Additionally, advanced eusocial species with clearly separated reproductive and nonreproductive castes are conceptually similar to multicellular organisms, and therefore, insights into the evolution of caste‐specific ageing in eusocial organisms might also apply to the evolution of ageing in reproductive and nonreproductive tissues in metazoans. Here, we present a model for the evolution of caste‐specific ageing in social insects based on William's antagonistic pleiotropy theory of ageing. Our results challenge the widely accepted explanation that lifespan divergences between castes in eusocial organisms can be explained by caste‐specific extrinsic mortality. Instead, reproductive monopolization by a single queen seems to be more important to explain these lifespan divergences, which can further increase if, for instance, limited resources need to be allocated between the queen and the workers.

In eusocial organisms, queens (and kings in termites) can have extraordinarily long lifespans of up to 30 years and live orders of magnitude longer than their workers (Keller 1998; Hartmann and Heinze 2003; Jemielity et al. 2005; Kramer and Schaible 2013a). As the castes of eusocial insects share nearly identical genomes, and caste polymorphism is based on caste‐specific gene expression patterns (Smith et al. 2008; Schwander et al. 2010), social insects provide excellent opportunities for studying the evolution of senescence or ageing (Keller and Jemielity 2006; Parker 2010; Kramer et al. 2016).

More than 20 years ago, Keller and Genoud (1997) proposed that the lifespan differences between social insect castes can readily be explained by the classical evolutionary theory of ageing, and their influential claim has been repeated ever since (Heinze and Schrempf 2008; Parker 2010; Kramer and Schaible 2013a). The classical evolutionary theory of ageing regards senescence as a nonadaptive process that evolved due to a declining force of selection with increasing age (Hamilton 1966), which can lead to the accumulation by genetic drift of deleterious mutations that have harmful effects in old age (Medawar 1952). As a further generalization of the classical theory, the antagonistic pleiotropy hypothesis suggests that this accumulation of mutations is even more likely if mutations have antagonistic effects, that is, positive fitness effects early in life and negative effects later in life (Williams 1957). In Williams’ seminal article, he also predicted that increased extrinsic adult mortality should lead to the evolution of shorter lifespans, that is, more rapid senescence, because increased extrinsic mortality decreases the likelihood that individuals reach old age and, hence, weakens selection against mutations expressed at old age (Williams 1957; Gaillard and Lemaître 2017; for objections on this prediction, see Caswell 2007; Wensink et al. 2017; Moorad et al. 2019). This logic, according to which intrinsic lifespan differences evolve due to differences in extrinsic mortality, has subsequently been adopted to explain the lifespan divergences between social insect castes (Keller and Genoud 1997). Social insect queens spend most of their time in well‐protected nests shielded from extrinsic mortality, while workers, in contrast, engage in risky behaviors like foraging and nest defense. Hence, it has been argued that the force of selection declines more rapidly with age in workers than in queens and consequently queens evolved longer lifespans than workers (Keller and Genoud 1997; Heinze and Schrempf 2008; Parker 2010).

However, the classical evolutionary models of ageing, which were developed for solitary organisms, do not necessarily apply to advanced eusocial insects (Kramer and Schaible 2013b; Kramer et al. 2016). In social insects, trade‐offs between fitness‐related traits, a defining premise of the antagonistic pleiotropy theory of ageing, do not only operate between traits expressed at different ages within the same individual, but also between individuals of different castes; for instance, if workers allocate more resources into somatic repair instead of sharing them with the queen, fewer resources will then be available for the queen, reducing her reproductive output or somatic repair at that point in time. Such trade‐offs result from indirect genetic effects, that is, effects of genes expressed in one individual on the phenotypes of other individuals (Wolf et al. 1998), which can cause antagonistic fitness effects between castes (Linksvayer and Wade 2005). Moreover, if fitness‐related traits are genetically correlated between castes, for example, due to shared use of gene regulatory networks, independent evolution of caste‐specific phenotypes could be restricted because of antagonistic selection between castes which prevents castes from achieving optimal trait values (Agrawal and Stinchcombe 2009; Hall et al. 2013; Holman et al. 2013; Pennell and Morrow 2013; Holman 2014; Holman and Jacomb 2017; Pennell et al. 2018).

Here, we present an individual‐based simulation model for the evolution of caste‐specific ageing in advanced eusocial insects in an antagonistic pleiotropy framework. The model represents a population of monogynous social insect colonies, each with a singly mated queen and sterile workers. Queens and workers possess gene regulatory networks that affect caste‐ and age‐specific survival probabilities and fecundities. All simulations start with identical queen and worker age‐specific survival rates, which can diverge during the simulation due to mutations in caste‐ and age‐specific regulatory genes, followed by selection and genetic drift. We explore (1) the effect of caste‐specific extrinsic mortality on caste‐specific ageing in social insects and (2) the quantitative effect of within‐caste and between‐caste antagonistic pleiotropy on the evolution of queen and worker lifespans.

## Materials and Methods

### LIFE HISTORY AND COLONY TURNOVER

We extended the individual‐based simulation model from Kramer et al. (2021). We assume a fixed‐size population of social insect colonies of a haplodiploid species in an individual‐based simulation model with discrete time steps. Colonies are monogynous and initialized with singly mated queens, all identical in their trait values in the first generation. The trait values are caste‐ and age‐specific survival and fecundities (20 age classes). Queens found colonies independently and are semiclaustral, which means that they have to forage in the colony founding phase, during which they are exposed to extrinsic mortality before they become shielded from it when they remain in the nest. We assume that queens and workers are equally successful foragers. The amount of resources foraged by a colony is then defined as

(1) R=z+w,

where *z* is the proportion of time that the queen spends foraging and *w* is the number of workers in the colony. During the complementary proportion of time 1‐*z*, the queen does not forage but instead lays eggs according to her age‐specific fecundity. The number of eggs laid by the queen per time step is

(2) $$E\;=\;f_{a}^{\left(Q\right)}\left(1-z\right),$$

where $f_{a}^{\left(Q\right)}$ is the age‐specific queen fecundity in age class a∈{1,2,3,…,20}. The average number of offspring produced by the colony at any given time step depends on the number of eggs laid by the queen (Eq. 2) and increases with the amount of resources (Eq. 1) foraged by the colony members on a per egg basis. Consequently, the number of offspring *F* is a product of the number of eggs *E* and the survival probability per egg which is an increasing function of the amount of resources per egg *R*/*E*, so that F=Eg(R/E), whereg(R/E) is an increasing function. We assume that g(x)=x/(1+x) with *x* = *R*/*E*. Then the average number of offspring is

(3) $$F\;=\frac{ER}{E+R}\;.$$

**Table 1:** Model parameters

Variable	Meaning	Value
*N*	number of colonies	1000
*T*	simulation time in time steps	10^6^
*k*	number of age classes	20
*h_ext_*	extrinsic hazard probability	0.0, 0.2
*μ*	mutation rate	0.005
*b*	mean mutational effect size	−0.2
*σ*	standard deviation mutational effect size	0.4
*β, γ, δ, ε*	partial correlations	−0.8
*m*	maximum fecundity per age class	5.0

The realized number of offspring is sampled from a Poisson distribution with a mean given by Equation (3). The proportion of time that a queen spends foraging at any time step is given by

(4) $$z\;=\;\max\left(0,\;1-\frac{1+w}{1+\sqrt{f_{a}^{\left(Q\right)}}}\right).$$

This equation has been derived by maximization of *F* with respect to *z*, assuming that queens optimize time allocation to foraging and egg laying.

Whenever the queen lays a diploid egg also one haploid egg is produced to ensure the presence of a sufficient number of males in the population such that all females can mate. Diploid eggs develop into workers or queens. Eggs destined to become workers or queens develop for one time step before they hatch. Queens leave the colony to occupy an empty colony spot (see below). Haploid eggs develop into males, which mature and die in the same time step because males are usually short lived and only function as gene carriers without trait expression in our model (for comparison: number of age classes and maximal lifespan of workers and queens is 20 time steps).

At the end of each time step worker and queen survival is checked. Individuals proceed to the next age class according to genetically determined intrinsic age‐specific survival probabilities and to an age‐independent extrinsic hazard probability *h_ext_*, which only foraging individuals are exposed to. The probability that an individual dies while foraging is defined as

(5) $$d_{ext}=\;1-{\left(1-h_{ext}\right)}^{z},$$

so that this probability depends on the extrinsic mortality probability and the time spent foraging. Thus, the probability that an individual survives the foraging trip is $s_{ext}=\;1-d_{ext}.\;$Workers always forage, thus, *z* = 1 and consequently for workers $d_{ext}=h_{ext}\;$. If the queen stays inside the nest, *z* = 0 and, thus, extrinsic mortality for the queen is $d_{ext}=\;0$. The survival of an individual is finally decided by sampling a Bernoulli distribution with parameter $s_{a}^{\left(C\right)}s_{ext}$, where $s_{a}^{\left(C\right)}$ is the age‐specific intrinsic survival probability of caste C∈{Q,W}. Additionally, individuals die when they reach the maximal age determined by the number of age classes.

If a colony is queenless, all colony members die. A new colony is founded by a mated queen, hatched from an egg from a random colony. New queens are sampled proportionally to the number of eggs in the colonies. A random male is sampled from all males in the population to mate with the new queen.

### GENETICS AND MUTATIONS

We assume that individuals carry a gene regulatory network which determines caste‐ and age‐specific survival and fecundity values. Queens express queen‐specific and workers worker‐specific trait values. A gene regulatory network, thus, affects 4*k* trait values (2 traits, 2 castes), where *k* is the number of age classes. These trait values are modelled as a vector of real‐valued numbers. Mutations affect regulatory genes of the gene network and can potentially have an effect on all castes, traits, and age classes. Diploid individuals carry two copies of the gene regulatory network, both coding for a vector of trait values. We assume that trait values interact additively to determine age‐specific survival and fecundity values. These values are transformed to caste‐ and age‐specific survival probabilities and fecundities using logistic functions (see Supporting Information).

Gene regulatory networks are mutated after transmission from the parents with probability *μ*. Antagonistic effects between mutations are encoded in a partial correlation matrix which is transformed to a mutational covariance matrix with which a multivariate normal distribution is initialized. The partial correlation matrix has dimensions equal to the number of trait values 4*k* and can be written as a block matrix

(6) $$\begin{array}{c} P\;=\left[\begin{array}{cc} \begin{array}{cc} WCWT & BCWT\\ BCWT & WCWT \end{array} & \begin{array}{cc} WCBT & BCBT\\ BCBT & WCBT \end{array}\\ \begin{array}{cc} WCBT & BCBT\\ BCBT & WCBT \end{array} & \begin{array}{cc} WCWT & BCWT\\ BCWT & WCWT \end{array} \end{array}\right]\;, \end{array}$$

where the square submatrices *WCWT* represent within‐caste within‐trait effects, *WCBT* represent within‐caste between‐trait effects, *BCWT* represent between‐caste within‐trait effects, and *BCBT* represent between‐caste between‐trait effects. Every submatrix of *P* consists of *k* ^2^ numbers. In all simulation scenarios, the submatrix *WCWT* is filled with values of 1 along the diagonal. If no antagonistic effects are assumed (baseline scenario), all other submatrices *WCBT*, *BCWT*, and *BCBT* are filled with zeroes. If antagonistic within‐caste within‐trait effects are assumed, the submatrix *WCWT* additionally contains delayed effects by five age classes. These effects are inserted five columns to the right from the diagonal, except in the last five age classes where the antagonistic effect would act after the maximum possible age of individuals. Submatrices *WCBT*, *BCWT*, and *BCBT* are filled with zeroes. We also ran simulations with effect delays of 3, 4, 6, 8, and 10 age classes, but only report the results for effect delays of five age classes in the main text because the effect delay hardly affected the results (Supporting information Fig. S1). If antagonistic within‐caste between‐trait effects are assumed, submatrix *WCBT* is manipulated with a delayed effect of five age classes, inserted five columns to the right from the diagonal, except in the last five age classes. Submatrices *BCWT* and *BCBT* are filled with zeroes. If between‐caste within‐trait effects or between‐caste between‐trait effects are assumed, submatrices *BCWT* and *BCBT*, respectively, are manipulated along the diagonal and submatrices *BCBT* or *BCWT*, respectively, are filled with zeroes.

After matrix transformation (see Supporting information), a 4*k*‐dimensional multivariate normal distribution, from which mutations are sampled, is initialized with the mutational covariance matrix. Mutations are sampled with a mean biased towards lower genetic values because most mutations are assumed to have negative effects. In addition to the results reported in the main text, we ran simulations with several different numerical values for the mutation bias (Supporting information Fig. S2).

### SIMULATIONS AND STATISTICS

**Figure 1:**
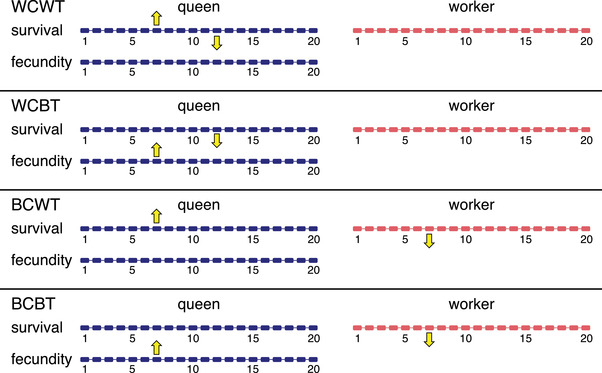
Antagonistic pleiotropy and correlations between fitness‐related traits in social insects. Yellow arrows show the positive (up arrow) or negative (down arrow) effect of mutations on survival and fecundity. Mutational effect sizes are drawn from a multivariate normal distribution with a negative mean (biased mutations). (WCWT) *Within‐caste within‐trait effects*. Mutations on traits are negatively correlated with mutations of the same trait at a later age class in the same caste. (WCBT) *Within‐caste between‐trait effects*. Queen fecundity is negatively correlated with queen survival at a later age class. (BCWT) *Between‐caste within‐trait effects*. Queen survival is negatively correlated with worker survival in the same age class. (BCBT) *Between‐trait between‐caste effects*. Queen fecundity is negatively correlated with worker survival in the same age class. Note that antagonistic effects do not act in relation to worker fecundity because workers are sterile.

In our model, each mutation can in principle affect each age‐ and caste‐specific trait (survival, fecundity), as determined by a mutational covariance matrix. We explored the following simulation scenarios where we set mutations to have different types of antagonistic effects which are likely to play a role in the evolution of social insect caste phenotypes (Fig. 1). *Baseline scenario*: mutations do not have antagonistic effects. *Within‐caste within‐trait effects*: a mutation has an antagonistic effect on the same trait expressed at different ages in the same caste. *Within‐caste between‐trait effects*: a mutation has an antagonistic effect on different traits within the same caste at different ages. *Between‐caste within‐trait effects*: a mutation has an antagonistic effect on the same trait at the same age in different castes. *Between‐caste between‐trait effects*: a mutation has an antagonistic effect on different traits in different castes at the same age. As we assumed that workers are sterile, antagonistic effects did not act in relation to worker fecundity.

We simulated each of these five simulation scenarios with identical extrinsic mortality probabilities for queens and workers of 0.0 or 0.2 to investigate one case where extrinsic mortality is absent in both castes and one case where extrinsic mortality is present for foraging individuals. We chose an extrinsic mortality probability of 0.2 to represent this second case. Extrinsic mortality probabilities of 0.1 or 0.3 delivered similar results (Supporting information Fig. S3). As we were interested in the effect of antagonistic pleiotropy on caste‐specific ageing, we assumed negative correlations of −0.8 between mutations affecting other trait values. However, we also simulated a range of other positive and negative effects to show how the effect size and direction affect our results under between‐caste effects (Supporting information Fig. S4). For each simulation scenario and extrinsic mortality, we ran 20 replicate simulations. Simulations were run for 10^6^ time steps, which represented a point where an evolutionary quasi‐equilibrium was reached. This was visually confirmed when mean trait values did not change much anymore. The model was implemented in C++, compiled with g++ and run on the Peregrine computer cluster of the University of Groningen.

At the end of each replicate simulation, we calculated the population mean age‐specific intrinsic survival probabilities and fecundities from the genomes of all living queens and workers in the population. We calculated evolved lifespans as the area under the population survival curve (life expectancy). Similarly, we calculated lifetime fecundity as the sum of the population's age‐specific fecundity values. This fecundity measure does consequently not include the probability with which an individual survives until a given age class.

Prior to statistical comparisons of evolved caste‐specific lifespans and fecundities in the different simulation scenarios and under the different extrinsic mortalities (0.0, 0.2), we transformed the evolved lifespans and fecundities to proportion data by dividing the evolved values by the maximum achievable lifespan (*k* = 20) or lifetime fecundity (k∗m=100), respectively. We ran beta regressions, separately for each caste and trait because all differences between castes were highly significant, using the function “betareg” from the R‐package *betareg* (Cribari‐Neto and Zeileis 2010). If significant differences were detected, we performed Tukey`s pairwise post‐hoc comparisons with a significance level of α = 0.05. Model output was analyzed in R 3.5.0 (R Core Team 2018) using the R‐packages *tidyverse* (Wickham et al. 2019), *cowplot* (Wilke 2019), *viridis* (Garnier 2018), *ggpubr* (Kassambara 2020), *emmeans* (Lenth 2018), and *multcomp* (Hothorn et al. 2008).

## Results

### LIFESPAN DIFFERENCES BETWEEN CASTES AND CASTE‐SPECIFIC EXTRINSIC MORTALITY

**Figure 2:**
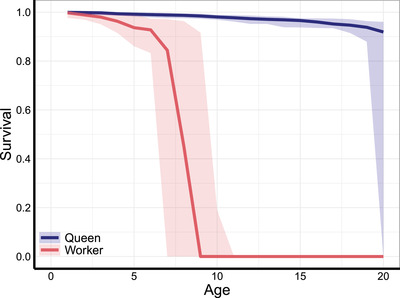
Evolved intrinsic survival curves of queens and workers in the absence of antagonistic pleiotropy and caste‐specific extrinsic mortality. Lines indicate medians of evolved age‐specific intrinsic survival probabilities and shaded areas the range across 20 replicate simulations. Parameter values as in Table 1. Note that workers age at a much faster rate than queens although extrinsic mortality is absent for both castes.

In different simulation scenarios, we assumed that mutations can have various antagonistic effects on survival or fecundity within castes or between castes (Fig. 1). In monogynous social insect colonies with sterile workers, significant lifespan differences between queens and workers evolved under all simulation scenarios and regardless of the presence of caste‐specific extrinsic mortality. Furthermore, lifespan differences between castes evolved in the absence of extrinsic mortality even if between‐caste within‐trait effects were not negative, that is, not antagonistic, but positively correlated, that is, positively correlated mutations of queen and worker survival probabilities (Supporting information Fig. S4). In all scenarios, queen and worker survival remained high until a certain age class where survival drops to nearly zero, typically at a much younger age for workers (Fig. 2).

### ANTAGONISTIC PLEIOTROPY AND QUANTITATIVE LIFESPAN DIFFERENCES BETWEEN CASTES

**Figure 3:**
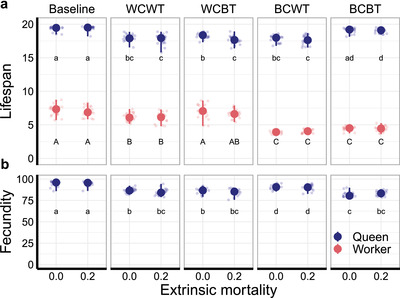
Evolved caste‐specific lifespans and fecundities for different antagonistic pleiotropy scenarios. For simulation scenario abbreviations, see Fig. 1. The baseline scenario does not include any antagonistic effects. Large dots indicate the median evolved queen and worker lifespan (A) and queen fecundity (B) and error bars the range across 20 replicate simulations. Transparent dots indicate population means of every single replicate simulation. All differences between castes were highly significant. We, therefore, ran separate beta regression models for the different castes (results in Supporting information Tables S1‐S3). Letters indicate statistically homogenous groups from Tukey`s pairwise post‐hoc comparisons within castes (α = 0.05).

Queen and worker lifespans and queen fecundities were less affected by the presence of caste‐specific extrinsic mortality than by antagonistic effects (Fig. 3; Supporting information Tables S1‐S3). Within‐caste within‐trait effects decreased queen lifespans, queen fecundities, and worker lifespans compared to the baseline scenario. Within‐caste between‐trait effects decreased queen lifespans and queen fecundities but had hardly any effect on worker lifespans. Between‐caste within‐trait effects decreased queen and worker lifespans. The effects on queen fecundities were comparably small. Between‐caste between‐trait effects decreased queen fecundities and worker lifespans but had only little effect on queen lifespans. In the two between‐caste antagonistic effect scenarios, worker trait values were more strongly affected than those of queens (Fig. 3; Supporting information Tables S1‐S3, Fig. S4).

## Discussion

Keller and Genoud (1997) hypothesized that evolved lifespan differences between social insect castes are explained by a caste differences in extrinsic mortality risk. We developed a model, tailored to advanced eusocial insects, in order to implement Williams’ antagonistic pleiotropy theory of ageing with the aim of deriving predictions for caste‐specific ageing. In our simulations, social insect colonies with a singly mated queen and sterile workers (which show the largest lifespan differences between castes among social insects; Keller and Genoud 1997; Kramer and Schaible 2013a) evolved large lifespan divergences regardless of whether caste‐specific extrinsic mortality occurred. Colonies of advanced eusocial insects, as those mimicked in our model, can be regarded as superorganisms with behaviorally and morphologically distinct, task‐specialized castes (Boomsma and Gawne 2018). Due to the difference in reproductive roles, workers, who do not directly reproduce, can only gain indirect fitness benefits. Inclusive fitness of both workers and queens is determined by the number of reproductive offspring produced. It has recently been shown that caste‐specific extrinsic mortality is neither necessary nor sufficient for the evolution of caste‐specific ageing because the strength of selection against deleterious mutations differs between castes irrespective of caste‐specific extrinsic mortality for two reasons: (1) selection against queen mortality is constant and maximal until the production of the first reproductive offspring which is typically delayed in social insects, whereas in workers the force of selection declines from eclosion onwards; (2) the strength of selection against queen mortality is higher than that on worker mortality because loss of the queen results in total collapse of colony reproductive output, while the loss of a worker cohort results in only partial failure as they can be replaced by younger workers (Kramer et al. 2021). These arguments, in which lifespan differences between social insect castes are not primarily explained by caste‐specific extrinsic mortality but by asymmetrical reproductive contributions of castes, do not imply that extrinsic mortality has no effect at all on the evolution of ageing in social insects. However, according to our results, extrinsic mortality is of relatively minor importance for explaining the difference in evolved lifespans found in queens and workers. These results were obtained under a density‐dependent probability that offspring found a new colony, and these conditions are favourable for extrinsic mortality to affect the evolution of ageing (Moorad and Promislow 2010). It is, therefore, likely that in different scenarios of density dependence, extrinsic mortality will also play a minor role in the evolution of lifespan divergences between queens and workers.

If reproductive division of labor is accompanied by caste‐specific differential resource allocation into maintenance versus reproduction or foraging, it is likely to further increase lifespan divergences between castes as indicated by our simulations with antagonistic effects between castes. These effects mainly decreased worker lifespans compared to the baseline scenario while the effect on queen lifespans was not as strong. This suggests that selection would favour allocation of limited resources to queens rather than to workers or that deleterious mutational effects are more likely to accumulate in workers than in queens when castes are exposed to antagonistic fitness effects. This distribution of deleterious effects between castes is likely to be influenced by reproductive skew. If reproductive skew is high, more resources would flow to queens and more deleterious effects would accumulate in workers than in queens, whereas at lower reproductive skew, resources would be more evenly distributed and deleterious mutational effects would accumulate more evenly in the castes (Pennell et al. 2018).

It could be that in early stages of an evolutionary transition to eusociality, caste antagonism plays a more prominent role than in advanced eusocial insects because in the absence of caste‐specific gene expression the same genes can be expressed in reproductives and helpers. In advanced eusocial species, in contrast, caste antagonism for some traits may have been overcome, for instance, by gene duplications, that might facilitate the evolution of caste‐specific gene expression, as indicated by extreme caste dimorphism (Linksvayer and Wade 2005; Pennell et al. 2018). Nevertheless, caste antagonism should still be prevalent in advanced eusocial species (Holman 2014) and furthermore indirect genetic effects can cause antagonistic effects between castes. If one caste evolves to invest more resources in somatic repair, these resources will become unavailable for immediate colony reproductive output or somatic repair of other individuals from other castes, thus, affecting the phenotype of the other colony members. This view, in which ageing arises from resource limitation and the resource allocation trade‐off between reproduction and survival, has been suggested by the disposable soma theory of ageing (Kirkwood 1977) which can be considered a more mechanistic version of the antagonistic pleiotropy theory of ageing. From a kin selection perspective, it is likely that workers evolve to allocate many resources to the queen instead of their own survival, as irreversibly sterile workers can only gain indirect fitness benefits by enhancing colony reproductive output (Bourke 2007) and these benefits can even be gained after the workers’ death. As predicted by the disposable soma theory of ageing (for an application to social insects, see Kramer and Schaible 2013b) and as shown by our between‐caste antagonistic effect scenarios, such resource allocation would increase lifespan divergences between castes.

Major evolutionary transitions, such as the transition to eusociality and the transition to multicellularity, have in common that helping individuals or cells forfeit their own reproduction to increase the reproductive output of a specialized reproductive caste or germ line (Maynard Smith and Szathmáry 1995; West et al. 2015). During such transitions, initial individual‐level selection can be reshaped into multilevel selection because of high relatedness between cooperating units (Grosberg and Strathmann 2007; Fisher et al. 2013). Ultimately, the unit of selection can be the group (or the new higher‐level individual; West et al. 2015) once a major evolutionary transition has passed an irreversibility threshold, characterized by the irreversible fixation of reproductive roles in early individual or cell development (Korb and Heinze 2004; Boomsma and Gawne 2018). Due to this irreversibility, advanced eusocial insect colonies, just as multicellular organisms, can be considered a higher‐level biological unit, the superorganism (West et al. 2015; Boomsma and Gawne 2018). The insights we gained for the evolution of ageing in social insects might, therefore, also apply to multicellular organisms in general (Kramer et al. 2016). Our model suggests that reproductive division of labor drives the evolution of caste‐specific ageing in advanced eusocial insects instead of caste or task‐specific extrinsic mortality associated with reproductive division of labor. Analogously to social insect castes, lifespans of cells in multicellular organisms can vary considerably and potentially the different reproductive contributions of the tissues might be the main evolutionary driver behind these lifespan divergences (Pen and Flatt 2021). Our model furthermore shows that especially antagonistic effects between castes can increase evolved lifespan divergences. These effects were weaker on queen lifespans than on worker lifespans. This indicates that there is a high fitness cost of resource allocation to workers and that selfish behavior of workers would decrease queen lifespan and fecundity, and thus, accelerate ageing of the queen and shorten colony lifespan. Similarly, intercellular conflict has been suggested to play an important role in ageing of multicellular organisms where, just as in a social insect colonies, the (super‐)organism has to function as a whole but where cells or individuals can have interests that deviate from supporting the system's productivity or functionality (Nelson and Masel 2017). In the case of multicellular organisms, such conflicts can lead to the selfish and cancerous reproduction of cells, deteriorating or ultimately causing the death of the organism (Nelson and Masel 2017).

Overall, our model shows that reproductive division of labor is more important to explain caste‐specific ageing in eusocial organisms than caste‐specific extrinsic mortality. Antagonistic effects quantitatively shape lifespan differences between queens and workers. Finally, these findings can potentially be extended to multicellular organisms and social insects might be an appropriate study system, not only to understand the evolution of ageing in eusocial organisms, but also for understanding the evolution of ageing along the transition to multicellularity.

## AUTHOR CONTRIBUTIONS

BHK, IP, and JJK designed the research. JJK did the programming and analyzed the model with support from IP and BHK. All authors contributed to the writing of the manuscript.

## DATA AVAILABILITY STATEMENT

Source code, data analysis scripts, and simulation results are available under https://doi.org/10.34894/DEVFRE.

## Supporting information


**Table S1**. Beta regression results for queen lifespans in the different simulation scenarios at extrinsic mortalities 0.0 and 0.2.
**Table S2**. Beta regression results for worker lifespans in the different simulation scenarios at extrinsic mortalities 0.0 and 0.2.
**Table S3**. Beta regression results for queen fecundities in the different simulation scenarios at extrinsic mortalities 0.0 and 0.2.
**Figure S1**. Evolved caste‐specific lifespans and fecundities for different effect delays under within‐caste within‐trait effects (WCWT).
**Figure S2**. Evolved caste‐specific lifespans and fecundities under different mutation biases under within‐caste within‐trait effects (WCWT).
**Figure S3**. Evolved caste‐specific lifespans and fecundities for different extrinsic mortalities under within‐caste within‐trait effects (WCWT).
**Figure S4**. Evolved caste‐specific lifespans and fecundities for different between‐caste within trait effect sizes (BCWT).
