# Supplementary material for: Antagonistic pleiotropy and the evolution of extraordinary lifespans in eusocial organisms
Source: Evol Lett. 2021 May 17;5(3):178–86. doi: 10.1002/evl3.230 (PMC8190452; doi:10.1002/evl3.230)
Supplement: Supplementary file 1 — Table S1. Beta regression results for queen lifespans in the different simulation scenarios at extrinsic mortalities 0.0 and 0.2. Table S2. Beta regression results for worker lifespans in the different simulation scenarios at extrinsic mortalities 0.0 and 0.2. Table S3. Beta regression results for queen fecundities in the different simulation scenarios at extrinsic mortalities 0.0 and 0.2. Figure S1. Evolved caste‐specific lifespans and fecundities for different effect delays under within‐caste within‐trait effects (WCWT). Figure S2. Evolved caste‐specific lifespans and fecundities under different mutation biases under within‐caste within‐trait effects (WCWT). Figure S3. Evolved caste‐specific lifespans and fecundities for different extrinsic mortalities under within‐caste within‐trait effects (WCWT). Figure S4. Evolved caste‐specific lifespans and fecundities for different between‐caste within trait effect sizes (BCWT). [file EVL3-5-178-s001.pdf]

Supplementary Materials

Title: Antagonistic pleiotropy and the evolution of extraordinary lifespans in eusocial organisms

Authors: Jan J. Kreider, Ido Pen, Boris H. Kramer

Transformation of trait values

Since the survival values are real-valued numbers, they are transformed to caste- and age-specific intrinsic survival probabilities using the logistic function

$$s_a^{(\mathrm{C})} = \frac{1}{1+\mathrm{e}^{-x_a^{(\mathrm{C})}}}, \quad (1)$$

where $x_a^{(C)}$ is the caste- and age-specific survival value. Survival values are initialized with a value of 3.2, resulting in an initial intrinsic survival probability of approx. 0.96 for all castes and age classes.

Fecundity values are transformed to caste- and age-specific fecundities using the logistic function

$$f_a^{(\mathrm{C})} = \frac{m}{1+\mathrm{e}^{-y_a^{(\mathrm{C})}}}, \quad (2)$$

where $y_a^{(\mathrm{C})}$ is the caste- and age-specific fecundity value and m is the maximum possible fecundity that can be reached by a queen. As workers are sterile, worker fecundities are never expressed. Fecundity values are initialized with a value of 0.5 which after transformation results in an initial fecundity of approx. 3.11 for all queen age classes.

Transformation of the partial correlation matrix to a mutational covariance matrix

The partial correlation matrix is transformed to a mutational covariance matrix as follows. First, all off-diagonal matrix elements are multiplied with -1. The partial correlation matrix is subsequently transformed to a correlation matrix with

$$R_{\mathrm{ij}} = \frac{(P^{-1})_{\mathrm{ij}}}{\sqrt{(P^{-1})_{\mathrm{ii}}(P^{-1})_{\mathrm{jj}}}}. \quad (3)$$

To obtain a mutational covariance matrix the correlation matrix is pre- and post-multiplied by a diagonal matrix of the same dimensions containing the standard deviations σ.

Table S1. Beta regression results for queen lifespans in the different simulation scenarios at extrinsic mortalities 0.0 and 0.2.

	**Estimate**	**SE**	**z**	**p-value**
(Intercept)	3.35	0.10	32.47	<0.001
WCWT	-1.17	0.12	-9.63	<0.001
WCBT	-0.97	0.12	-7.86	<0.001
BCWT	-1.25	0.12	-10.40	<0.001
BCBT	-0.27	0.14	-1.93	0.05
extrinsic mortality 0.2	0.11	0.15	0.72	0.47
WCWT:extrinsic mortality 0.2	-0.21	0.17	-1.21	0.23
WCBT:extrinsic mortality 0.2	-0.46	0.17	-2.62	0.01
BCWT:extrinsic mortality 0.2	-0.17	0.17	-1.01	0.31
BCBT:extrinsic mortality 0.2	-0.27	0.19	-1.37	0.17

Table S2. Beta regression results for worker lifespans in the different simulation scenarios at extrinsic mortalities 0.0 and 0.2.

	**Estimate**	**SE**	**z**	**p-value**
(Intercept)	-0.58	0.03	-18.05	<0.001
WCWT	-0.24	0.05	-5.17	<0.001
WCBT	-0.05	0.05	-1.07	0.29
BCWT	-0.83	0.05	-16.54	<0.001
BCBT	-0.67	0.05	-13.76	<0.001
extrinsic mortality 0.2	-0.04	0.05	-0.86	0.39
WCWT:extrinsic mortality 0.2	0.01	0.07	0.20	0.85
WCBT:extrinsic mortality 0.2	-0.04	0.06	-0.55	0.58
BCWT:extrinsic mortality 0.2	0.07	0.07	1.02	0.31
BCBT:extrinsic mortality 0.2	0.04	0.07	0.51	0.61

Table S3. Beta regression results for queen fecundities in the different simulation scenarios at extrinsic mortalities 0.0 and 0.2.

	**Estimate**	**SE**	**z**	**p-value**
(Intercept)	2.91	0.09	30.83	<0.001
WCWT	-1.06	0.11	-9.40	<0.001
WCBT	-1.08	0.11	-9.54	<0.001
BCWT	-0.61	0.12	-5.13	<0.001
BCBT	-1.41	0.11	-12.88	<0.001
extrinsic mortality 0.2	0.01	0.13	0.08	0.94
WCWT:extrinsic mortality 0.2	-0.12	0.16	-0.77	0.44
WCBT:extrinsic mortality 0.2	-0.13	0.16	-0.82	0.41
BCWT:extrinsic mortality 0.2	-0.089	0.168	-0.530	0.596
BCBT:extrinsic mortality 0.2	0.090	0.155	0.577	0.564

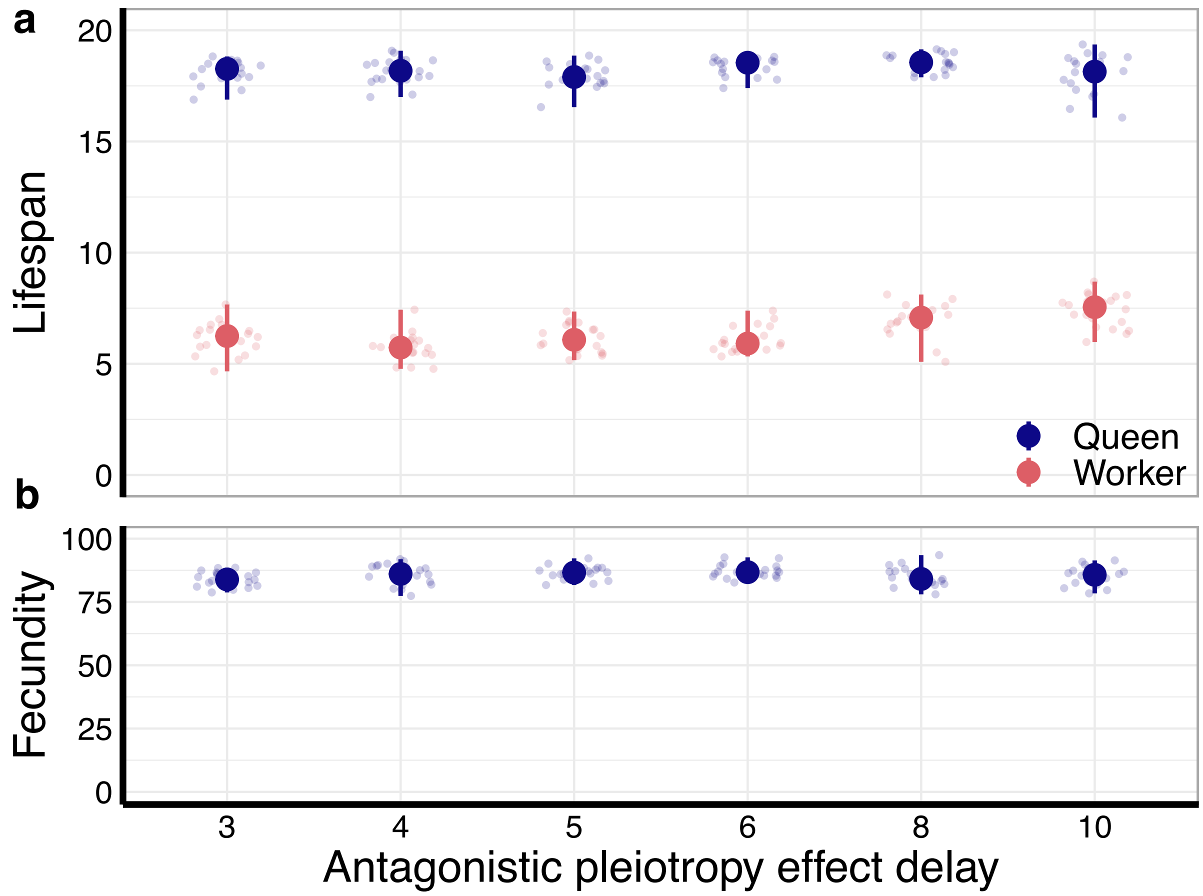

Figure S1. Evolved caste-specific lifespans and fecundities for different effect delays under within-caste within-trait effects (WCWT). Large dots indicate the median evolved lifespan (A) and fecundity (B) and error bars the range across 20 replicate simulations. Transparent dots indicate population means of every single replicate simulation. Results for effect delays of 3, 4, 5 or 6 show hardly any differences. If the effect delay was 8 or 10, worker lifespans were longer compared to shorter delays. In these cases, workers were hardly affected by antagonistic pleiotropy because such delayed effects were never realized within a worker's lifespan (compare with worker lifespans in baseline scenario). Extrinsic mortality = 0.0.

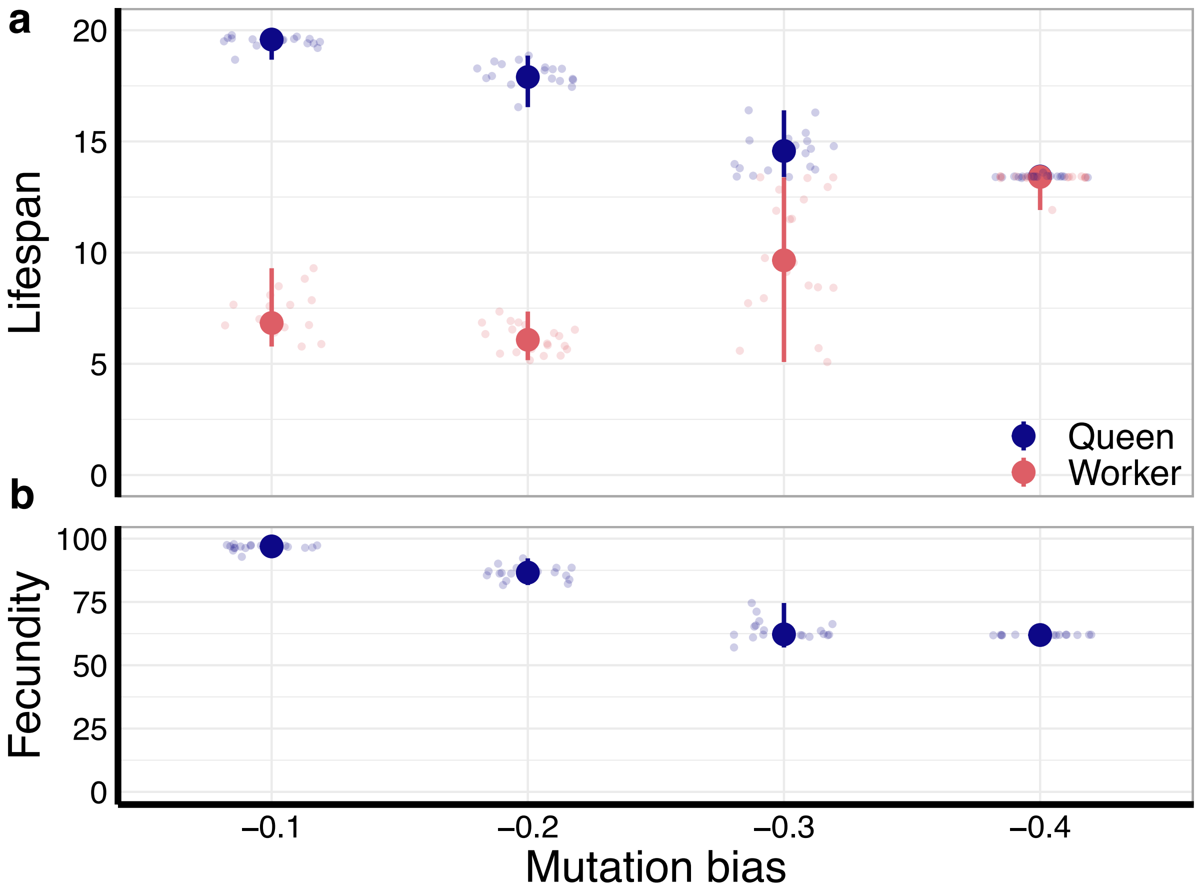

Figure S2. Evolved caste-specific lifespans and fecundities under different mutation biases under within-caste within-trait effects (WCWT). Large dots indicate the median evolved lifespan (A) and fecundity (B) and error bars the range across 20 replicate simulations. Transparent dots represent population means of every single replicate simulation. If mutation bias was too strong, selection was overwhelmed by mutations with negative fitness effects, and consequently lifespan divergences between castes diminish. We therefore chose a value of -0.2 for our simulation. Effect delay = 5.

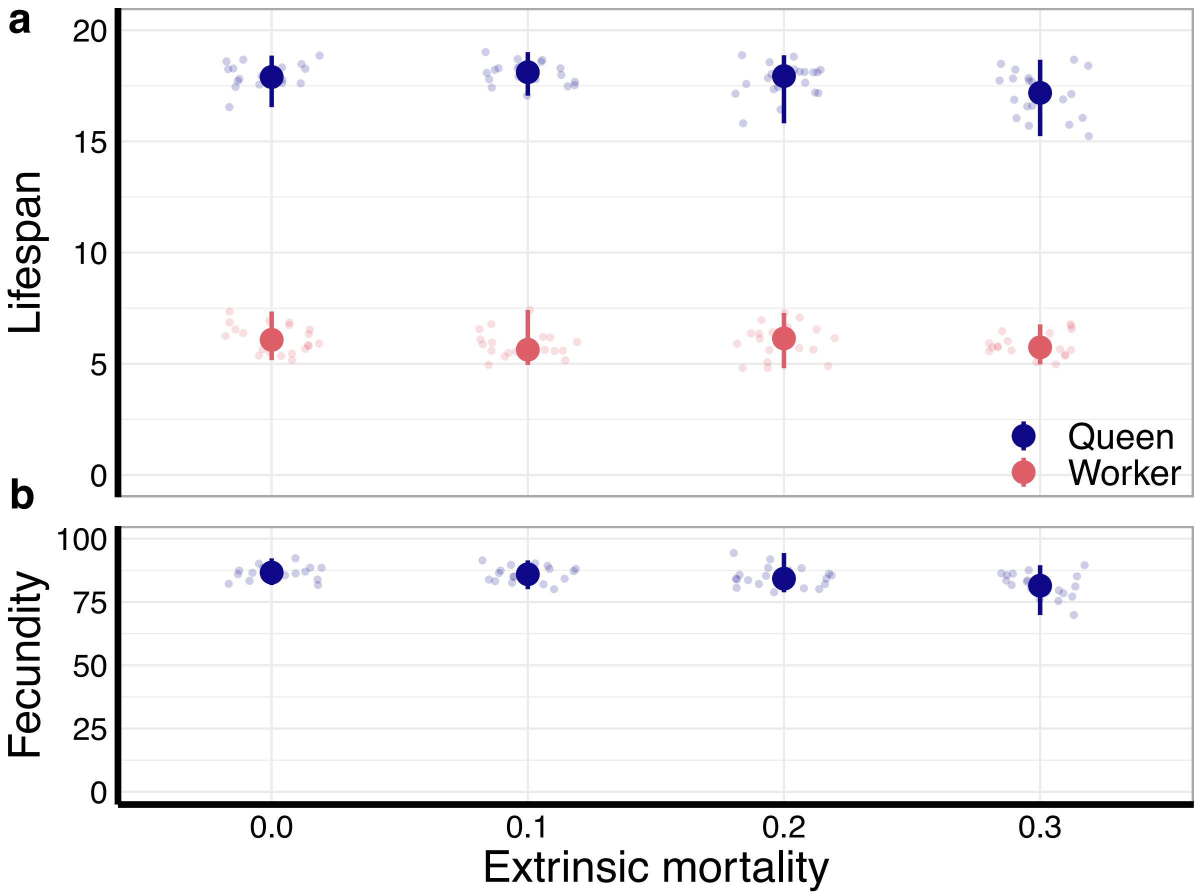

Figure S3. Evolved caste-specific lifespans and fecundities for different extrinsic mortalities under within-caste within-trait effects (WCWT). Large dots indicate the median evolved lifespan (A) and fecundity (B) and error bars the range across 20 replicate simulations. Transparent dots represent population means of every single replicate simulation. There were hardly any differences between queen and worker lifespans under the different extrinsic mortality probabilities. Effect delay = 5.

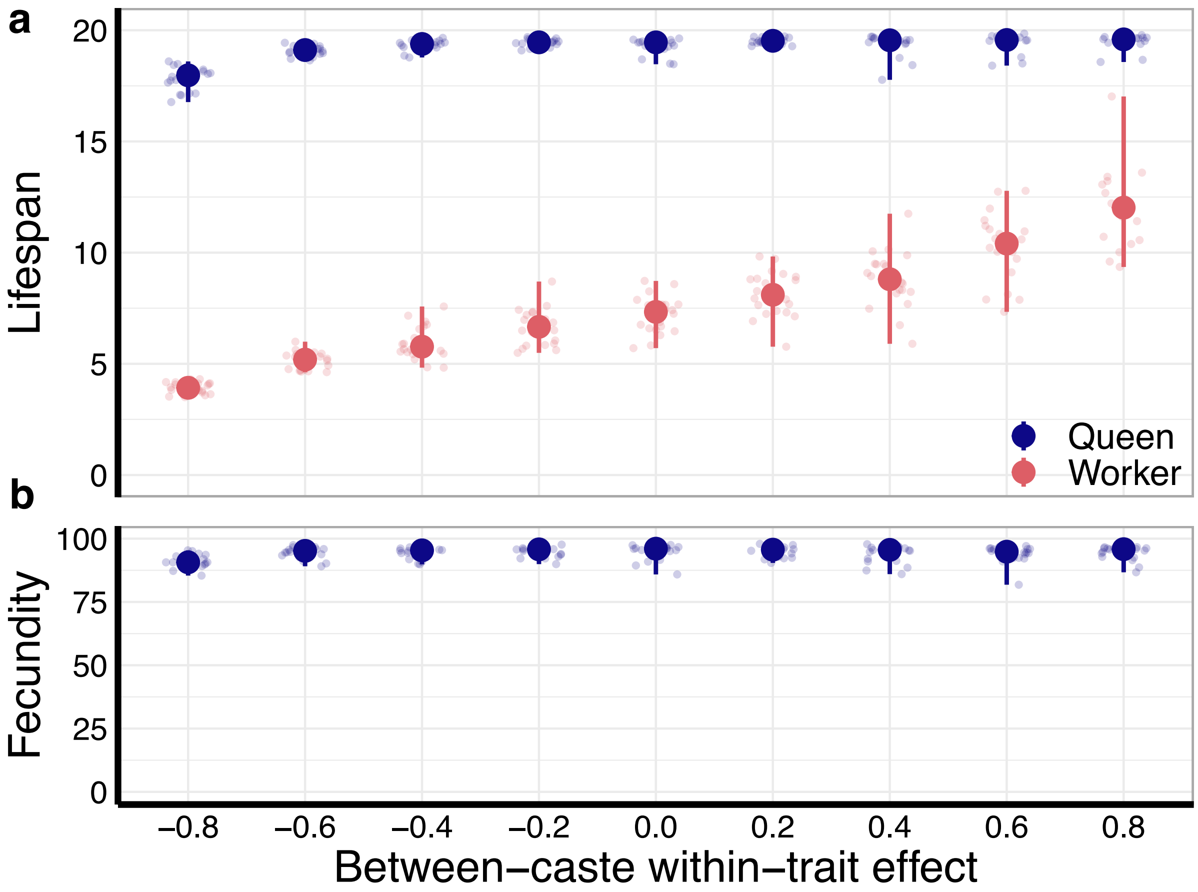

Figure S4. Evolved caste-specific lifespans and fecundities for different between-caste within-trait effect sizes (BCWT). Large dots indicate the median evolved lifespan (A) and fecundity (B) and error bars the range across 20 replicate simulations. Transparent dots are population means of every single replicate simulation. Lifespan divergences between castes get larger when the between-caste correlation gets smaller or more negative. Even under positive between-caste effects lifespan differences between castes evolved. Extrinsic mortality = 0.0.
